# A scoping review on laboratory surveillance in the WHO Southeast Asia Region: Past, present and the future

**DOI:** 10.7189/jogh.13.04028

**Published:** 2023-04-21

**Authors:** Vidushi Goel, Silvy Mathew, Nachiket Gudi, Anil Jacob, Oommen John

**Affiliations:** 1 The George Institute for Global Health, New Delhi, India; 2 Public Health Evidence South Asia, Department of Health Information, Prasanna School of Public Health, Manipal Academy of Higher Education, Manipal, Karnataka, India; 3 The George Institute Services, New Delhi, India; 4 Prasanna School of Public Health, Manipal Academy of Higher Education, Manipal, Karnataka, India

## Abstract

**Background:**

The South-East Asia (SEA) region bears a significant proportion of the world’s communicable disease burden. The onset of the COVID-19 pandemic has further affected the situation. A well-established laboratory-based surveillance (LBS) can reduce the burden of infectious diseases. In light of this, the review collated the existing literature on LBS system in the region and the modifications adopted by the surveillance systems during the pandemic.

**Methodology:**

We followed the guidelines for scoping review as prescribed by Arskey and O’Malley. We comprehensively searched three databases (PubMed, Scopus and CINAHL) and supplemented it with grey literature search. The screening of the articles was conducted at the title and abstract followed by full-text screening. This was followed by data extraction using a pre-tested data extraction tool by two independent reviewers. The results were presented narratively.

**Results:**

Including 75 relevant articles and documents, we compiled a list of surveillance systems. A shift from paper to dual (paper and electronic) modalities was identified across the countries. This largely low- and middle-income countries (LMIC) area face challenges in reporting, resources, and collaboration-related issues. While some countries have well-established National Reference Laboratories; others have more private than public-owned laboratories. Given the COVID-19 pandemic, modifications to the existing laboratory capacities to enable real-time surveillance was identified. Laboratory capacity complemented with genomic surveillance can indubitably aid in disease detection and control. Limitations due to inaccessible government portals, and language barriers are acknowledged. This review identified a comprehensive list of surveillance systems in the region, challenges faced in using these surveillance systems and inform the decision makers about the benefits of integrating fragmented surveillance systems.

**Conclusion:**

Regionally and nationally integrated genomic and laboratory surveillance systems justify capital investments, as their payoffs rationalise such costs owing to economies of scale over time. Further, as data flows are harmonized and standardized, algorithm- and computing-based pattern recognition methods allow for targeted and accurate disease prediction when integrated with, potentially, climate and weather systems data. Trained human resources are a sine qua non to optimize such investments, but in the medium to long run, such investments will buttress initiatives in other arenas at the regional level.

Owing to its biodiversity, and being in a largely tropical zone, the World Health Organization’s South East Asia Region (WHO SEAR) consists of eleven countries and hosts many epidemiological hotspots [1]. These countries have a high burden of infectious diseases outbreaks as well as emerging and re-emerging diseases including those of zoonotic origin [2]. Evidence shows that certain areas of the world are more likely to experience the emergence of new infectious diseases than others, and these are termed global “hotspots” for emerging infectious diseases (EID) [3]. Some areas of the WHO SEAR that are a part of these global hotspots are parts of Bangladesh and India (the Indo-Gangetic plains) and regions of Thailand along with boundaries of Myanmar (the Mekong River basin). The region has not only been a host to Nipah virus (NiV), Crimean-Congo Haemorrhagic fever (CCHF), and Avian Influenza A (AI A) (H5N1) but has also contributed to the rapid spread of the recent Coronavirus Disease (COVID-19) [4].

As a region with both high population and the need for economic development, factors such as sanitation, changes in the interactions between humans, wildlife, and nature, shifting land use patterns, and antimicrobial resistance have all contributed to disease outbreaks in the region [5]. These outbreaks and the incurred losses in economic terms and human lives, can be progressively reduced by integrating laboratory surveillance systems with existing networks, thus enhancing the robustness of the existing surveillance system.

A robust and well-connected surveillance network helps in quickly identifying outbreaks dispersed over wide geographic areas. Surveillance systems in the past have been catering to the needs of vertical disease programs but have not been successful in containing diseases [6-8]. Observing the failures of these vertical disease surveillance programs, the World Health Organization (WHO) has advocated an integrated approach to disease surveillance to target multiple diseases with existing resources [6-8].

Successful detection, characterization, and tracing of disease transmission are attributes of an efficient public health laboratory system [9]. Laboratory-based surveillance is one of the pillars in the notification and monitoring of infectious disease trends [10]. Timely reporting of these events confirmed through laboratory diagnosis contributes to a well-informed disease containment strategy.

Apart from laboratory surveillance, another significant aspect that can aid in controlling any pandemic, like COVID-19, is the genomic surveillance. It is a process or strategy by which the entire course of an outbreak, from the disease spread to its evolution can be understood and used to inform effective control strategies to contain further transmission [11]. Despite the identified benefits of genomic surveillance in the containment of various diseases such as tuberculosis, malaria, HIV, food-borne pathogens, and / or antimicrobial resistance, evidence has suggested that the capacity for genomic surveillance remains low in other low-income regions like Africa as well. With the advent of COVID-19, the demand for building a laboratory system that is complemented by a robust genomic surveillance capacity has become even more pertinent [12].

While the existing scholarly literature highlights the role and functioning of surveillance systems, there is a paucity in the literature of studies demonstrating the importance of consolidating the laboratory-based surveillance systems in the WHO SEAR region and the modifications that have taken place in the existing system during this pandemic [13,14]. Thus, this scoping review was carried out with a dual objective of identifying different laboratory-based surveillance systems in the WHO Southeast Asia Regional Office (SEARO) region and documenting the modifications adopted during this pandemic with an aim to identify novel approaches, innovations and best practices that could be shared and scaled up across the region and in other LMICs.

## METHODS

Given these research objectives, a scoping review was regarded as the best method to identify the existing surveillance systems and their adaptations during the COVID-19 pandemic. This type of review is usually adopted to clarify working definitions, conceptual boundaries and to map the current findings of any topic of interest within a particular field [15]. The preferred methodological framework for conducting this review is the Arskey and O’Malley’s framework [15]. A protocol was also developed before the commencement of the study. All the authors were involved in identifying the research question, designing the protocol and any deviations from the protocol are reported later in the article. The study followed the six sequential steps outlined in the guidelines to effectively address our research objectives. The review was reported according to the PRISMA extension for scoping reviews (PRISMA-Scr) checklist [16].

### Stage 1: Identifying the research question

The research questions were constructed to understand the various laboratory linked surveillance systems within the region and further detail on how these systems were used during the COVID-19 pandemic within the WHO SEARO region. Following are the research questions: What are the various laboratory-linked surveillance systems within the countries of the WHO SEARO region? How have these identified laboratory-linked surveillance systems been used during the COVID-19 pandemic and to highlight, if so revealed, potential gaps?

### Stage 2: Identifying relevant studies

A comprehensive search strategy was developed by NG, VG and SM to identify the relevant literature. The grey literature was searched using government websites / portals to identify suitable keywords. These keywords informed in developing an extensive search strategy. The search was conducted between May 18 and 22, 2021 on three databases: (1) PubMed (NCBI), (2) Scopus (ELSEVIER), and (3) CINAHL (EBSCO) by the search team (VG, SM) and further validated by NG. The search was restricted to articles from WHO SEAR countries (by geography), those published in the English language, and between January 2010 and April 2021. The search was restricted to the last decade as the number of articles with the key word “laboratory surveillance” published in this time period are gradually increasing. We also conducted an additional search, with the same search string, on May 31, 2022, to identify any potential new study relevant for this review, and one study was included.

### Search strategy

The identified keywords were related to laboratory linked, surveillance system, and names of the WHO SEAR countries. Various synonyms of these keywords were then used in conjunction with Boolean operators like AND / OR to form an appropriate search string and adapted to each database. The search string used for PubMed (NCBI) is presented in **Table 1**.

**Table 1:** PubMed search string

PubMed search string
((laboratory linked OR laboratory based OR laboratory based OR Laboratory network) AND (“Surveillance” OR “monitoring” OR “Surveillance System*” OR “Disease Surveillance System*” OR “Early warning” OR “Early detection”)) AND ((“WHO SEARO” OR “South East Asia Region” OR “SEAR” OR “WHO SEAR”) OR (((Bangladesh OR India OR Bhutan OR Indonesia OR Maldives OR Myanmar OR Nepal OR Sri Lanka OR Thailand OR Timor-Leste)) OR (DPRK OR Democratic Republic of Korea OR North Korea OR Democratic People's Republic of Korea)).

### Stage 3: Study selection

All identified studies from the database search were imported into EndNote X9.3.3, Clarivate Analytics [17], US, where duplicates were removed. The remaining articles were imported to Rayyan (a screening software) [18], where the two authors (VG and SM) independently screened all the titles and abstracts for relevant studies. The screening was conducted in two sequential stages where the Title-Abstract (Ti-Ab) stage was followed by full-text screening.

The articles in this stage were sorted based on three categories: include, exclude and maybe. Any disagreements between the two authors were resolved in discussion with the third authors (NG and OJ). As a second step, the full texts of potential studies were screened by the two authors (VG and SM), and the reasons for excluding the full-text articles were recorded and are presented in Table S1 in the **Online Supplementary Document**. The final set of articles were discussed among all authors to rule out any disagreement through consensus. The goal of the scoping review was to provide an overview of the available literature; therefore, the articles were included regardless of quality.

### Stage 4: Charting the data

For the final set of included records, two authors (VG and SM) reviewed and extracted all the relevant information using a data extraction tool devised. The domains identified for charting the data were: Author’s last name, year of publication, study setting (country), study design, characteristics of the study, laboratory component, and genomic component. The data extraction tool is provided in Tables S3 and S5 in the **Online Supplementary Document**. The identified grey literature was also charted using the same data extraction tool. Additionally, the names of surveillance systems identified in the articles were also listed (mentioned in the results section) and information on the same were extracted.

### Stages 5 and 6: Collating, analysing, summarizing, and reporting the results

We conducted data analysis of the collated information to present the results narratively, using frequencies and percentages. The data was managed using MS Excel (Microsoft Inc, Seattle WA, USA) and Google Sheets. The findings were later supplemented with tables and graphs. We employed a narrative synthesis approach to provide an overall summary of all the findings of the studies.

## RESULTS

### Search results

**Figure 1:**
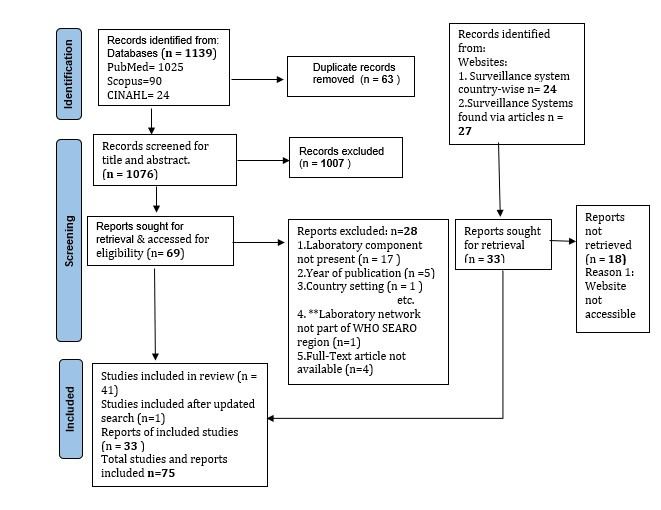
PRISMA Flow-diagram.

An initial country-wise grey literature search was conducted on government websites, and that yielded 24 reports closely related to laboratory surveillance. Data from government portals for Timor-Leste, Myanmar, and Indonesia were not accessible due to technical and language issues. On completing the grey literature search, a comprehensive search was conducted on three databases (PubMed, Scopus and CINAHL), resulting in 1139 articles and duplicate records were removed. The remaining 1076 records were screened based on titles and abstracts, resulting in the selection of 69 records, which were assessed for eligibility in full text. Of these, 41 articles were considered eligible for the study and 28 articles were excluded. The reasons for exclusion are provided in the PRISMA flow diagram (**Figure 1**). The second search, conducted on May 31, 2022, yielded one relevant article, which was later included in this review.

Additionally, during the data charting stage of the 42 eligible articles, names of the surveillance systems were extracted and subsequently, grey literature was searched for 24 laboratory surveillance systems. We could access the websites for only nine surveillance systems. Finally, 75 records (including articles and grey literature) were selected for this scoping review.

### Characteristics of articles found

#### Geographical distribution of studies

**Figure 2:**
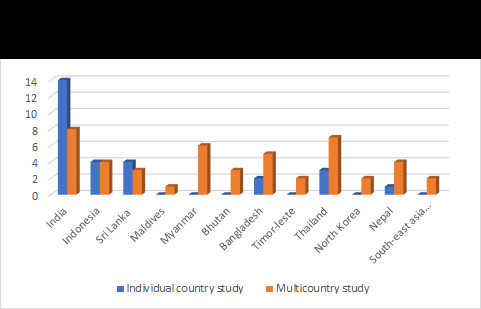
Distribution as per the geographical context.

As depicted in **Figure 2**, most of the studies were conducted in India (15 studies representing Indian geography alone and eight being multi-country studies). This was followed by Indonesia and Sri Lanka for single country studies, and Thailand and Myanmar for multi-country studies. We also found that no single country studies were conducted in the Maldives, Timor-Leste, Myanmar, North Korea, and Bhutan. Also, there were two studies that were conducted in the SEAR as a whole.

#### Year-wise distribution of studies

The maximum number of studies, that is, seven were published in 2016 [19-25] and 2018 [13,26-31], whereas fewer studies were published in 2010 [32], 2011 [33], and 2015 [34] with one article each (**Table 2**).

**Table 2:** Distribution of studies as per year of publication

Year of publication	No. of studies (n)
2010	1 [32]
2011	1 [33]
2012	2 [35,36]
2013	5 [7,37-40]
2014	2 [41,42]
2015	1 [34]
2016	7 [19-25]
2017	4 [43-46]
2018	7 [13,26-31]
2019	5 [47-51]
2020	4 [52-55]
2021	3 [56-58]
Total	42

#### Type of study designs

For most of the articles, study designs were not clearly specified, we decided to classify them as “potential cross-sectional study” and “not clearly identified”. A list of these studies is presented in **Table 3**.

**Table 3:** Type of study designs

Type of study design	No. of articles (n)
Cross-sectional study	11 [22-24,27,34,43,44,53,55,58,59]
Potential cross-sectional study	6 [21,24,28,40,48,49]
Secondary study design	2 [39,50]
Cohort study	1 [38]
Review	9 [13,30,32,33,35,46,47,49,57]
Report	1 [31]
Mixed-method study	1 [42]
Qualitative study	1 [56]
Not clearly identified	10 [19-21,25,27,36,37,41,52]
Total	42

#### Characteristics of grey literature found

To gain a thorough understanding of the laboratory surveillance systems in the WHO SEARO region, a comprehensive grey literature search was also conducted through government websites and portals of all the WHO SEAR countries. The grey literature search results for all the countries, with the year of implementation of the systems is mentioned as under in **Table 4**. Surveillance systems for Timor Leste, Myanmar, and Indonesia could not be identified due to inaccessible government websites / portals and information published in language other than English.

**Table 4:** List of surveillance systems identified through grey literature search

Country	Year	Surveillance system
**Sri Lanka**	Not clear	Notifiable Disease Reporting System
	1991	National Poliomyelitis Eradication Initiative Acute Flaccid Paralysis Surveillance Programme
	2019	National Action Plan for Prevention and Control of Dengue
**Nepal**	1997	Surveillance of Communicable Disease Program
	2019	National Malaria Surveillance Guidelines
	2019	National Guidelines on Prevention, Management and Control of Dengue
	2016	National Antimicrobial Containment Action Plan
**Bhutan**	2014	National Early Warning, Alert and Response Surveillance
	2014	National Guidelines for Management of Rabies
	2014	National Guidelines for Management of Leprosy
	2018	National Action Plan on Antimicrobial Resistance
**Bangladesh**	2009	Web-Based Priority Communicable Disease Surveillance
	2012	Hospital Based Rotavirus Intussusception Surveillance
	2007	Acute Meningoencephalitis Surveillance
	2017	National Action Plan Antimicrobial Resistance Containment
**India**	2018	Integrated Disease Surveillance Program module of Integrated Health Information Platform
	2017	National Tuberculosis Elimination Program
	2017	National Action Plan on Antimicrobial Resistance
**Maldives**	2015	Communicable Disease Surveillance System
	2009	National Influenza Pandemic Preparedness Plan
	2019	National Antimicrobial Containment Policy
**North Korea**	2014	Infectious Disease Surveillance System
	2018	National Strategic Plan on Antimicrobial Resistance
**Thailand**	Not clear	National Laboratory system

### Characteristics of the surveillance system

#### Type of surveillance

**Figure 3:**
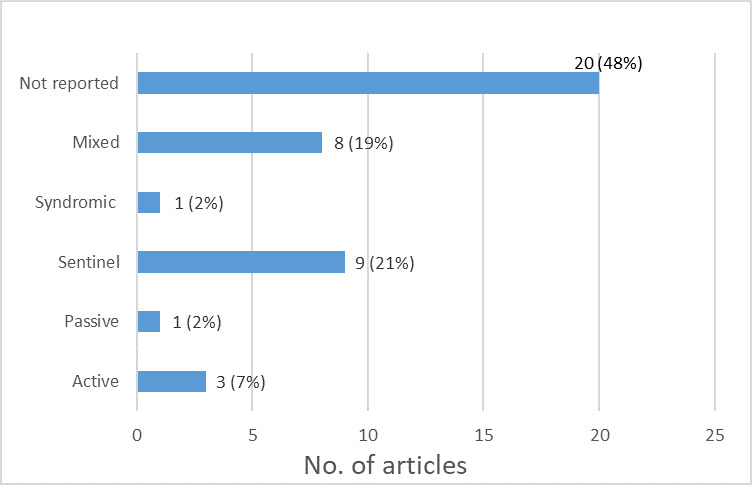
Distribution of studies as per the type of surveillance.

The most common type of surveillance identified in the articles was sentinel surveillance, reported in nine (12%) studies [19,20,26,28,31,49,50,54,55] out of the 42 included articles (**Figure 3**). This was followed by a mixed type of surveillance, comprising a combination of active, passive, sentinel, or survey, which were mentioned in eight (19%) studies [21,22,30,34,35,41,48,59]. However, in 20 (48%) articles [13,21,23-25,27,29,33,36,39,40,42-44,46,47,52,56-58], the specific type of surveillance was not indicated.

#### Mode of surveillance

**Figure 4:**
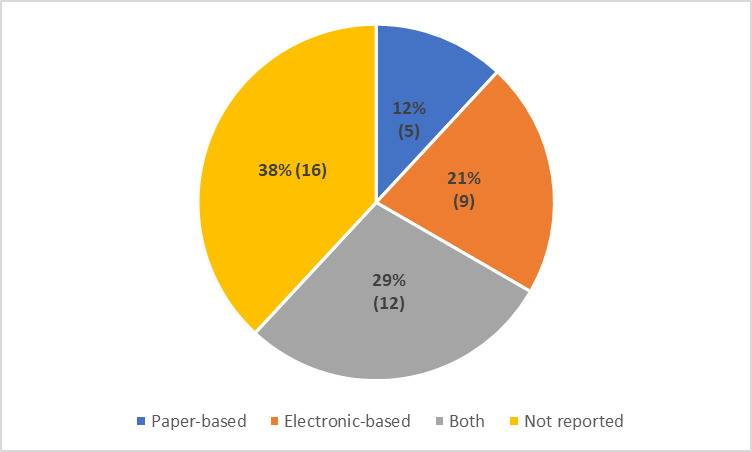
Distribution of studies as per mode of surveillance.

From the 42 studies, five (12%) [22,23,37,40,53] reported a paper-based mode of surveillance, whereas nine (21%) [24,25,29,38,47-49,52,55] reported an electronic mode of surveillance, followed by 12(29%) [19,21,26,28,33,43,50,54,56,57,59,60] studies which used both paper and electronic based surveillance. Additionally, 38% articles [13,20,27,30,31,34-36,39,41,42,44-46,51,58] did not specify any mode of surveillance explicitly (**Figure 4**).

#### Level of surveillance

**Figure 5:**
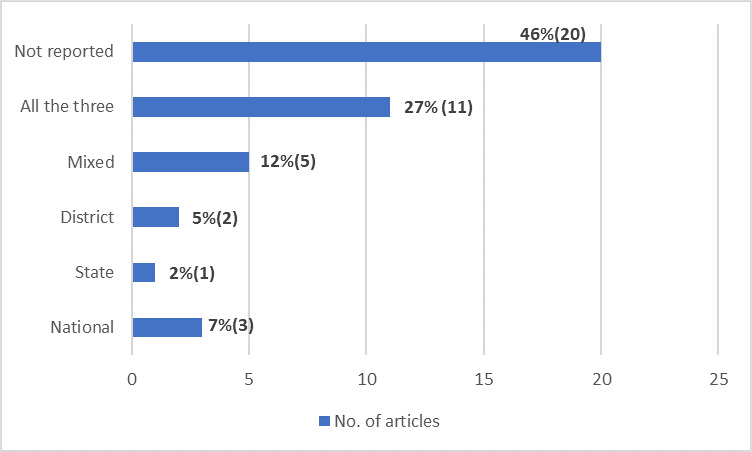
Distribution of studies as per the level of surveillance

Nearly half (46%)of the studies [13,20,21,23,26,28-31,39,40,42-45,51-53,56,58] did not clearly specify the level of surveillance (national, state or district); however, 27% of studies [32,35,36,41,48-50,54,55,57,59] reported surveillance at all three levels of the system. More than one level of surveillance was mentioned in five (12%) studies [19,22,24,37,46]; therefore, we categorised these as being a mixed level of surveillance (**Figure 5**).

#### Diseases captured

**Figure 6:**
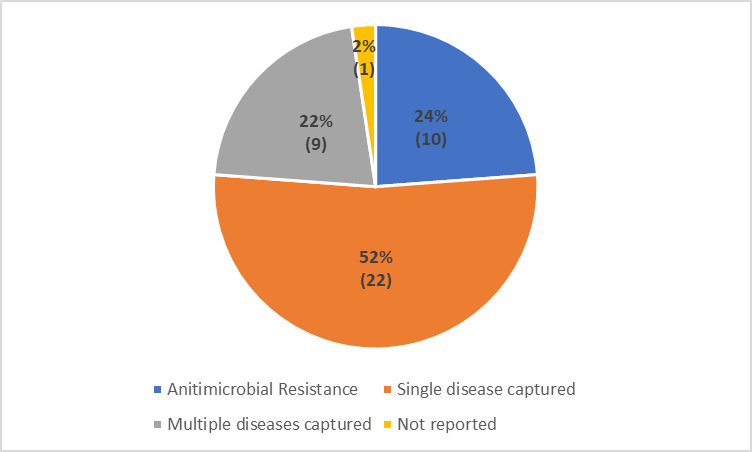
Distribution of studies as per the diseases captured.

Single disease was captured in 22 (52%) of the studies [20,22-27,31,34,35,37,41-44,48-51,54,55,57], as depicted in **Figure 6**, whereas nine (22%) studies [13,19,21,32,33,39,40,45,59] targeted multiple diseases. Antimicrobial resistance was reported as the focus of the surveillance system in 10 studies [28-30,36,39,46,47,52,53,58]. Although the review intended to describe the type of surveillance system by classifying them as vertical or integrated, the information could not be retrieved, as none of the articles reported on the same.

### Description of laboratory surveillance capacity

The information acquired from both peer-reviewed articles and grey literature was compiled to describe the country-wise utilization of laboratory surveillance in the WHO SEAR.

#### Bangladesh

In Bangladesh, Nipah virus surveillance was conducted by the means of the Acute Meningo-Encephalitis surveillance system, which also incorporates surveillance for other diseases such as Japanese encephalitis (JE), dengue, and other bacterial causes for encephalitis [61]. Furthermore, evidence on the feasibility of using an already existing laboratory network for polio and measles surveillance, in the detection of JE [19]. However, laboratory-supported surveillance for vaccine-preventable bacterial diseases will require substantial technical and financial support to enhance local diagnostic capacity. Furthermore, to detect JE and bacterial meningitis (BM) by syndromic surveillance, an attempt was also made to further expand an established laboratory network for polio and measles surveillance in the country [19]. Although the current diagnostic capacity was found to be feasible for detection of JE, it was not the same for BM, due to differences in laboratory staff and testing methodologies [19]. Similarly, a pre-existing system of laboratory-based surveillance for invasive bacterial-vaccine Preventable diseases was expanded to include the surveillance for enteric fever [45].

#### Bhutan

Bhutan has the National Early Warning and Response System, supplemented by individual laboratory-based sentinel surveillance systems for diseases such as influenza-like illness (ILI), diarrhoea, dengue, multi-drug resistance tuberculosis, and other diseases [62]. Bhutan’s National Action Plan on Antimicrobial Resistance, published in 2018, highlights the importance of data generated through laboratory surveillance and therefore, the action plan intends to further increase the laboratory capacity in the country to fulfil its objective [63].

#### India

In India, laboratory capacity for surveillance exists for measles [37], tuberculosis [64], rotavirus disease [23,24], polio / acute flaccid paralysis (AFP) [13,33] and antimicrobial resistance (AMR) [65]. It has established referral laboratory networks for AMR and tuberculosis [64]. For AMR, India also has a system called Indian Council of Medical Research (ICMR) Antimicrobial resistance surveillance system (iAMRSS) that aims to capture standardized data on AMR from small laboratories to have comprehensive nationwide data [47]. The country also has established animal laboratories for AMR surveillance, although these laboratories are not currently integrated with the human laboratories [58]. Because the veterinary and environmental sectors have limited capability for antibiotic susceptibility testing, the majority of data on antimicrobial resistance originate from human sources [58]. India was among the first countries to start environmental surveillance (ES) for AFP in 2001 [13]. Environmental surveillance has been utilising the already existing Global Polio Laboratory Network (GPLN) in the SEAR region by increasing its capacity [13].

Among the integrated systems, the Integrated disease surveillance program (IDSP) [32] and integrated plan on chikungunya and measles were found [66]. The IDSP module of Integrated Health information platform (IHIP) is a robust surveillance system in India in which data collection from the laboratory is a key component [67]. The data are collected using three forms S (syndromic), P (presumptive), L (laboratory). In this system, laboratory reporting is done with the aid of the reporting form “L” [67]. The system also comprises a strong referral laboratory network. The integrated programme on measles and chikungunya, on the other hand, utilizes laboratories for early case detection and comprises 13 apex referral laboratories with advanced diagnostic tests and facilities for disease detection [66].

Similar to IHIP data collection forms, data capture forms (DCFs) were utilized in India, Bangladesh and Pakistan in a population-based surveillance study, called Aetiology of Neonatal infections in South-East Asia (ANISA) study [21]. Recognizing the importance of high-quality laboratory performance, these countries developed an efficient system for data tabulation by the means of these DCFs [21]. These DCFs were used to capture relevant specimen-related information followed by real-time transference of the data to ANISA databases of the respective sites by the laboratory personnel. This data are regularly transferred to the central server every week [21].

Additionally, a review conducted in India reported that there were sentinel or hospital-based surveillance systems, which utilized reference laboratories such as the National reference laboratory and Virus research and diagnostics laboratory network [49].

#### Indonesia

Our searches highlighted Indonesia’s laboratory-based congenital rubella syndrome (CRS) [44] and avian influenza (H5N1) surveillance [41]. For the H5N1 disease, in 2007, to enhance the early detection capacity in the country, influenza network laboratories and the national influenza centre of Indonesia were established for case detection and surveillance [35,41]. Additionally, the regional animal laboratories were prepared to detect influenza cases in animals. The early warning and reporting system (EWARS) was another surveillance system identified in Indonesia. Although the system uses laboratory data for surveillance, a qualitative study on the same suggested that laboratory strengthening is required in the country. The laboratory network comprises two reference laboratories in the capital city, Jakarta, and eight regional laboratories in eight provinces of Indonesia [56].

#### Maldives

Maldives conducts disease surveillance by utilising a National Reference Laboratory (NRL), as prescribed in its pandemic preparedness plan [68]. The identified NRL was Indira Gandhi Memorial Hospital, which is used for testing ILI The regional laboratories were found to be not suitable for testing due to lack of biosafety standards, so their role was only recognized for sample collection and transport to the reference laboratory [68].

#### Myanmar

Myanmar is also part of ES alongside India, Bangladesh, Thailand, and Nepal, for AFP surveillance utilizing the same laboratory network in the region as mentioned above [13]. A hospital-based surveillance study in the Thailand-Myanmar border conducted a case-based surveillance using a laboratory for detection and confirmation [40].

#### Nepal

Laboratory surveillance for diseases such as malaria [69] and dengue [70] were identified. An integrated disease surveillance system named EWARS was found, which has been collecting laboratory data for surveillance since 1996 [71]. Furthermore, for AMR, within the country, the National Antimicrobial Resistance Surveillance Network conducts surveillance utilizing National Public Health Laboratory along with its associated referral network laboratories [36,72].

#### Democratic People’s Republic of Korea (DPRK) / North Korea

Information on North Korea’s laboratory utilization was scarce. For AMR surveillance, Pyongyang Medical College was found to be recognized as an NRL for the country. All the provincial laboratories reported their data collection to the aforementioned NRL [73].

#### Sri Lanka

As part of the national Melioidosis surveillance program, a laboratory-supported network of surveillance exists [27]. For invasive Melioidosis, laboratory-based case finding was conducted in coordination with Western Australian Public Health Laboratory and WHO [25]. Also, to further enhance melioidosis surveillance in Sri Lanka, molecular technology in clinical laboratories was introduced as a part of WHO’s laboratory capability-building program [25]. A laboratory-enhanced sentinel surveillance for dengue is also active in the country. This surveillance was coordinated with Genetech Research Institute Colombo, a private sector non-profit research institute, which acted as an interim testing laboratory in the first year of the project [22]. For lymphatic filariasis, the country has a laboratory network equipped with decent resources, collaboration and research support [42], although it was not found to be the same for the Notifiable Disease Reporting System of Sri Lanka [74].

#### Thailand

Thailand has a National Laboratory System, which encompasses private, public, regional laboratories and an established reference laboratory [75]. The system is equipped to conduct six out of 10 WHO-defined core tests and also detect priority EIDs. The National Institute of Health is the designated reference laboratory for the country. We found vertical disease surveillance systems for pneumonia (Severe pneumonia surveillance system-SevPn) and AFP [13,48]. Both were reported to use laboratories for surveillance; however, details for the extent of laboratory use in SevPn could not be found. On the other hand, for AFP, similar to India, ES in collaboration with laboratories has been in place since 2016 [13]. Additionally, under the one health approach, animal health and environmental laboratory networks are linked in the country [75].

#### Timor Leste

No information for Timor Leste could be found from the scholarly literature and while conducting the grey literature search, we could not acquire any information, as the links to the government portal were non-functional at the time.

### Global Surveillance Systems that were active in the region

Studies highlighted a varying status of laboratory-based AMR surveillance in the countries of the SEAR region. Out of 11 member states, nine countries have national reference laboratories for testing sensitivity to antibiotics, and seven reported a good laboratory system support for their tertiary care hospitals [46] although there is a potential lack of utilization of common international standards to generate comparable data on laboratory functioning. Also, private laboratories were found to be more as compared to government-owned (public) laboratories in the region [53]. Software like the WHO’s NET and other software to monitor resistance patterns were not popular in the region, despite its free availability and simplicity [45]. Moreover, a comprehensive AMR surveillance system was only found in Thailand with plans to further expand the network [52].

The global networks utilizing laboratory-based surveillance identified to be active in WHO SEAR countries were the following: the WHO’s Gonococcal Antimicrobial Surveillance Programme [39], the Global Influenza Hospital Surveillance Network [51], the Global Antimicrobial Surveillance System [30], Automated tool for Antimicrobial resistance Surveillance System (AMASS) (an offline data collection software for AMR) [52], the Enhanced Gonococcal Antimicrobial Surveillance Programme [28], and the Global Influenza Surveillance and Response System (GISRS) [76].

### Genomic surveillance

Information on genomic surveillance was scarce; a few studies [2,21-25,43,45,67] reported an active status of genotyping for disease pathogens, along with regular laboratory diagnostic procedures. Mostly, genotyping was found to be done as part of surveillance-based research studies, to determine the genetic trends of the disease. The genomic surveillance component was identified as a part of established disease surveillance networks such as the Global Polio Laboratory Network [13], National Melioidosis Surveillance Program (Sri Lanka) [25], laboratory-supported surveillance for CRS (India) [31], Indian National Rotavirus Surveillance Network [24], National Antimicrobial Surveillance Network (Nepal) [36], National Guidelines on Prevention, Management and Control of Dengue (Nepal) [70] and iAMRSS [47].

### Gaps and challenges

**Figure 7:**
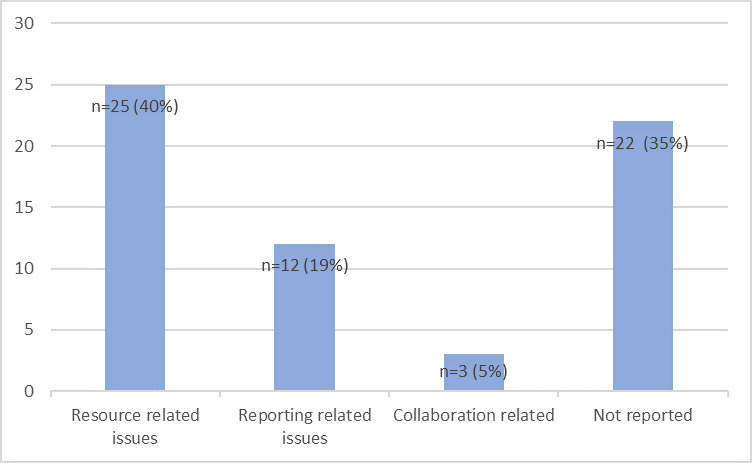
Distribution of studies as per gaps and challenges identified.

The gaps and challenges identified through the articles were categorized into domains such as collaboration-related, reporting-related, resource-related challenges, respectively, and those that were not reported. Weak laboratory infrastructure, delayed reporting and lack of trained professionals were issues commonly mentioned across studies (**Figure 7**). There were 22 articles which did not report any gaps and challenges, assuming it to be beyond their scope. A complete list of laboratory related gaps and challenges identified across the studies is provided in **Table 5**.

**Table 5:** List of gaps and challenges mentioned in the articles

Resource related	Reporting related	Collaboration related
Weak laboratory capacity with lack of adherence to international standards [19,21,35,46,48,53,56,58,59]	Limited IT infrastructure [53]	Poor collaboration with the private sector [43]
Lack of trained personnel [19,33,35,41,56,58]	Delayed and under-reporting of the diseases [13,19,30,33,40,48,55,57,59]	Lack of connectivity between secondary and tertiary laboratories [19]
Logistic issues [21,41,59]	Limited mobile and internet connectivity [56]	Lack of partner engagement [30]
Storage infrastructure [59]	Lack of proper documentation quality [42]	
Underutilization of resources [35,41]		
Limited funding [30,42,56,58]		

### Developments during COVID-19 and lessons learnt

The review showed varying levels of modifications adopted by the countries across the region, during COVID-19 pandemic. In India, laboratory surveillance for COVID-19 is coordinated by the ICMR, in partnership with VRDL, Department of Science and Technology, Department of Biotechnology (DBT), Indian Council of Agricultural Research (ICAR), (Council of Scientific & Industrial Research, Defence Research & Development Organisation, Ministry of Human Resource Development, medical colleges, and private laboratories [57]. Initially, laboratory testing, and surveillance were performed by 78 selected national laboratories, which was later expanded using the existing laboratory network by developing standard protocols and utilization of an online portal for reporting [55].

However, for Sri Lanka, a pre-existing virology laboratory was identified as the central testing laboratory to successfully respond to COVID-19 [77]. As of June 2020, the laboratory strategy for COVID-19 highlighted three types of epidemiological investigations, which had an essential laboratory (testing) component involved. The strategy focuses on sentinel surveillance from Out-Patient Departments (OPDs), random sampling from high-risk areas, and seroprevalence studies in defined study populations [77]. Additionally, GeneXpert machines, which were typically used for Tuberculosis diagnosis, were also used for testing in COVID-19 surveillance [77].

Modifications were also made to the existing WHO’s Global Influenza programme (GIP), wherein a strategy was adopted to simultaneously support influenza surveillance and SARS-CoV2 monitoring [78]. This was conducted by employing the national influenza sentinel surveillance system within the member states, in the context of COVID-19 pandemic. Recommendations on data sharing and laboratory diagnosis were some of the noteworthy directions rendered by WHO [78]. Issues such as overwhelmed laboratories and lack of human resources capacity for testing, member states were suggested to use Multiplex kits for testing both Influenza and SARS CoV-2, by using the WHO recommended algorithm [77].

## DISCUSSION

The ongoing COVID-19 pandemic expounded the importance of a well-connected laboratory-based surveillance system in many ways, and that is what sparked our interest to explore the current situation of the existing systems in the WHO SEA region. This ScR identified various surveillance systems within the region as they constantly battle multiple levels of socio-technical challenges. Delayed reporting, incomplete data, and lack of human resources to manage data were commonly highlighted data reporting challenges. A study from the African region presented similar findings where resource constraints have been commonly observed [12]. Issues related to resource limitations can be reduced by digitizing the surveillance systems, but the process should be well informed based on existing digital literacy levels [79] and cultural sensitivity, as appropriate, to the needs of health care workers [80]. The process of using electronic devices with automated reminders for data collection can reduce errors in data entry, ensure completeness and timeliness of reporting. Electronic reporting can minimize delayed reporting and improve data quality when compared to paper-based reporting [81].

We found that there are nine NRLs from 11 countries in the region. Although the countries have referral laboratories associated with the NRLs, drawbacks such as the lack of uniform standards for the functioning of these laboratories coupled with infrastructural limitations exists [46]. A comprehensive regional network for AMR surveillance could not be found in the SEAR, while a regional network (MR LabNet) exists for measles and rubella (MR) [82]. In 2017, the network comprised 40 laboratories (one regional reference laboratory, 25 NRLs, and 14 sub-national laboratories) [82]. In addition to these, the WHO coordinates an integrated global laboratory network to support surveillance for selected Vaccine Preventable Diseases [83]. These laboratory networks provide capacity-building and infrastructure support to all public health laboratories of member states. Sustained funding is a key challenge for resource-constrained settings in prolonging operations [83]. A good example of a well-established regional laboratory network (RLN) is WHO’s European Region (EUR), which comprises operational laboratory networks for 17 diseases for the entire region [84]. Such integrated RLNs can be beneficial in the WHO SEAR.

With the rise in Antimicrobial Resistance (AMR) alongside zoonotic diseases among humans, the importance of a well-coordinated, multi-sectoral and multi-disciplinary response was recognized by many international organisations that led to the introduction of “One Health” as a key initiative to counteract emerging infectious diseases (EIDs) [85,86]. From the SEA Region, Thailand (which operates cross-linked environmental and animal health laboratory systems) [75] and India launched a network of laboratories for AMR surveillance in the animals and fisheries sectors in collaboration with Food and Agriculture Organization (FAO) and the ICAR [87]. The requirement for such a coordinated response is further reinforced with the onset of the ongoing pandemic.

The 2021 Global Health Security Index (GHSI) has also pointed out that all countries are “dangerously unprepared” for future pandemics, which could potentially be worse than the present day COVID-19. The infrastructural issues such as shortage of reagents were even faced by developed countries such as the USA, along with developing countries of SEAR, highlighting insufficient laboratory capacity to mitigate the pandemic response across the globe [88-90]. In SEAR, there is considerable variation in capacities: only Thailand ranks among the top five countries in the world, in terms of laboratory surveillance and detection capacity [91]. Thailand’s enhanced capacity in the region may well be attributed to its economic stature compared to other SEAR countries and the country is also one of three countries leading the world’s efforts to strengthen national laboratory systems [92].

The SEA Region has more private laboratories than public laboratories [93]. Mandating the reporting of communicable diseases that are endemic to the region for both the private and public facilities can further strengthen such surveillance systems. Passive private participation has been a challenge in India for reporting data in other portals as well. Collaborative efforts can minimize this issue and in turn enhance the capacity of surveillance systems [94].

Another challenge identified was the lack of adequate Internet connectivity and mobile network in the region. According to the World Bank’s statistics, less than 30% of the population use the internet in India, Bangladesh, and Timor-Leste, demonstrating a low internet penetration, resulting in limited accessibility to the internet [95]. To overcome these network-related challenges, more systems like AMASS should be built, wherein, the reporting could be done irrespective of internet connectivity [52]. Another approach to tackle the issue could be by engaging with the local community as demonstrated by a San Francisco-based non-profit, “Ending Pandemics”. They have engaged local organizations, citizens and software developers of lower-income countries, and have established that technology-enabled surveillance systems in coordination with other active stakeholders can help in effectively reducing disease transmission. The creation of such sustainable, integrated, and user-friendly technologies can benefit resource-limited countries by reducing the installation expenses of massive infrastructures to aid in disease detection capabilities, and response.

The COVID-19 pandemic also highlighted the role of genomic surveillance and identified whole genome sequencing (WGS) as the most important advancement in infectious disease laboratory technology [96]. Due to the current pandemic, capacity to sequence genome increased and more than 68% of countries were identified to report genome sequence data. In order to sustain and strengthen the current progress, WHO has developed a global genomic surveillance strategy [97]. Global initiative on sharing all influenza data are another noteworthy initiative that provides open access genomic data for all viruses [98]. All these data sharing platforms are pivotal to understand the evolution and mutation of viruses to effectively control the spread during epidemics or pandemics. Thus, such collaborative actions from global, regional or national agencies are the need of the hour to boost the capacities and efficiency of disease detection and response in resource constrained settings.

The advent of EID in the WHO SEAR highlighted the importance of continuous surveillance to aid rapid response, which can be achieved by strengthening genomic surveillance capacities [99]. Our review highlighted the scarcity of information on integrating genomic surveillance into laboratory surveillance. Genomic surveillance is mostly done as part of the surveillance-based studies to determine genetic trends; however, few established surveillance networks in WHO SEAR incorporated genomic surveillance capacity as an integral part of the broader surveillance system [13,24,25,31,36,47,70]. A COVID-19 weekly report for the WHO SEAR reported that, out of 11 countries of the SEAR, three countries have the capacity to perform genome sequencing at their national public health laboratories, while four countries have capacities to conduct genomic surveillance beyond designated national laboratories as well (research institutes, universities, and private laboratories contribute to this capacity in these countries). On the other hand, the remaining four countries of the SEAR do not have in-country sequencing capacity and have been supported by WHO’s SEARO, in association with COVID-19 reference laboratories / WHO collaborating centres [100]. Clearly, a robust surveillance system consisting of both laboratory and genomic components, at the regional level is vital. Since most SEAR countries are LMICs, setting up such a regional level surveillance system will be cost-effective due to economies of scale. We propose the following recommendations to enhance the capacity of existing laboratory surveillance systems in the region.

### Recommendations

Adopting a strategy such as integrating hospital-based surveillance (public and private) with mortality surveillance and national laboratory-based surveillance would be pivotal in informing decision-making at various levels. Integrated systems such as these could provide data on positivity, testing rates and caseloads – vital parameters for policy-scalable decision-making and the necessary allocation of resources for public health purposes. Belgium has employed such an approach and has made rapid strides in responding to the pandemic [101-103]. Standardizing indicators and data collection forms using platforms such as the WHO Integrate Data Platform (WIDP) that facilitate response mechanism would be imperative for such endeavours [104]. The need for capacity building is highlighted owing to the passive participation from private facilities and poor documentation practices. There are regional field epidemiology programs that have trained medical officers thereby enhancing their practical skills in health care management, use of computers and epidemiology [105]. Such programs must be tailored to staff who are involved in testing and tracing contacts during an outbreak. Such training activities may further reduce turnaround time between testing and declaring results. In order to facilitate timely data-driven decision in situations where time is scarce, there is a need to evaluate surveillance systems for the usability, timeliness and completeness of laboratory surveillance systems [106]. Hu et al. emphasise “cross-jurisdictional data to support information sharing, analysis, and visualization in public health” [107]. With the emergence of this pandemic, the felt need for harmonization of surveillance data, the need for comprehensive yet flexible data sharing policies has also been increasing [108]. The 74th World Health Assembly (WHA) in 2021 further emphasized the need for building international cooperation for strengthening alert systems and data sharing [108]. We strongly suggest that the same is ratified in the upcoming pandemic treaty with member nations being signatory to it as the treaty will provide for stronger enforcement mechanisms [109]. With multiple surveillance systems already active in the region, we recommend integrating these at the national and, most importantly, at the regional level, thereby envisioning the possibility of end-to-end surveillance. A similar approach is advocated by the WHO in their interim guidance [110,111]. Sharing of data between meteorological and public health departments can aid in real time prediction [112]. Employing techniques of pattern recognition by considering both climate and disease are deemed necessary and they aid in predicting the course of outbreaks [113]. Such approaches cannot only minimize the cost of establishing new surveillance systems and dedicated human resources but also strengthen collaborative response activities within the region. Lastly, more research is required on the laboratory surveillance capacity, usability and the socio-technical aspects of these surveillance systems in the region, as compiling adequate information for this review was extremely challenging.

### Strengths and limitations

We conducted an extensive search, for both peer-reviewed articles and the grey literature, to capture maximum information on the laboratory-based surveillance systems in the WHO SEAR countries. At first, a grey literature search was conducted to identify relevant keywords, followed by a database search to retrieve articles. Later, a second grey literature search was conducted for the individual surveillance system names identified through the articles chosen for the review.

While searching for information, language was a major limiting factor (English being the preferred language) because of which information for certain countries like Myanmar and Indonesia could not be extracted. For countries like Thailand and Timor-Leste grey literature could not be found due to inaccessible government websites. Therefore, limited information was retrieved for the aforementioned countries.

### Deviations from protocol

Although the review was informed using a protocol (not registered) developed a priori to systematically approach the scoping review, there were a few deviations. First, during the full-text stage of the review, we decided to capture data on genomic surveillance, as a few articles covered this aspect of surveillance, and this has been a priority area of research within the SEARO. Second, the two-step grey literature (apart from informing the search strategy) search was not planned during the protocol development, it was incorporated during the review implementation stage, to get a comprehensive picture of laboratory surveillance in the region.

## CONCLUSION

This review was conducted with an intent to emphasize the significance of laboratory surveillance in the early detection and control of diseases in the region. The incurred losses due to the disease outbreaks, can be progressively reduced by integrating laboratory surveillance systems in the region, thus enhancing the robustness of the existing surveillance system. A network of well-connected referral laboratories can corroborate that laboratories are not overwhelmed during epidemics or pandemics. Laboratory surveillance capacity complemented with genomic surveillance can further aid in successful disease detection and control. Installation of such integrated systems can help overcome the current challenges of limited funding, inadequate laboratory infrastructure, reporting related issues and logistical issues in the region. Investing in such digitally advanced, integrated systems can be challenging financially for a predominantly LMIC region like SEAR in the short run. However, such investments definitely can be amortized by reducing the societal costs of disease burdens in the long run.

## Additional material


Online Supplementary Document
