# Supplementary material for: A scoping review on laboratory surveillance in the WHO Southeast Asia Region: Past, present and the future
Source: J Glob Health. 2023 Apr 21;13:04028. doi: 10.7189/jogh.13.04028 (PMC10119808; doi:10.7189/jogh.13.04028)
Supplement: Online Supplementary Document [file jogh-13-04028-s001.pdf]

Online Supplementary Documents

Table S1: List of articles excluded during full-text screening with reasons for exclusion

S.No.	Excluded Articles	Reason for Exclusion
1.	Antibiotic resistance in Bangladesh: A systematic review	Laboratory surveillance system not highlighted
2.	Cholera incidence among patients with diarrhoea visiting National Public Health Laboratory, Nepal	Full-text article not accessible
3.	Detection of the emergence of mcr-1-mediated colistin-resistant Escherichia coli and Klebsiella pneumoniae through a hospital-based surveillance in an oncology centre in eastern India	Laboratory surveillance system not highlighted
4.	Expanding poliomyelitis and measles surveillance networks to establish surveillance for acute meningitis and encephalitis syndromes--Bangladesh, China, and India, 2006-2008	Year of publication not as per inclusion criteria
5.	Expansion of syndromic vaccine preventable disease surveillance to include bacterial meningitis and Japanese encephalitis: evaluation of adapting polio and measles laboratory networks in Bangladesh, China and India, 2007-2008	Year of publication not as per inclusion criteria
6.	Field- and laboratory-based active dengue surveillance in Chennai, Tamil Nadu, India: observations before and during the 2001 dengue epidemic	Year of publication not as per inclusion criteria
7.	Genetic sequencing for surveillance of drug resistance in tuberculosis in highly endemic countries: a multi-country population-based surveillance study	Laboratory surveillance system not highlighted
8.	Genomic surveillance for hypervirulence and multi-drug resistance in invasive Klebsiella pneumoniae from South and Southeast Asia	Laboratory surveillance system not highlighted

9.	Health and Demographic Surveillance Systems Within the Child Health and Mortality Prevention Surveillance Network	Demographic surveillance system. Infectious disease and Laboratory surveillance system not covered.
10.	Hospital based surveillance and molecular characterization of rotavirus in children less than 5â€¯years of age with acute gastroenteritis in Nepal	Laboratory surveillance system not highlighted
11.	A hospital based surveillance of metallo-beta-lactamase producing gram negative bacteria in Nepal by imipenem-EDTA disk method	Laboratory surveillance system not highlighted
12.	Hospital-based surveillance for Japanese encephalitis at four sites in Bangladesh, 2003-2005	Laboratory surveillance system not highlighted
13.	Hospital-based zoonotic disease surveillance in Bangladesh: design, field data and difficulties	Laboratory surveillance system not highlighted
14.	IASM: A System for the Intelligent Active Surveillance of Malaria	Country setting not as per inclusion criteria
15.	Implementation of surgical site infection surveillance in low- and middle-income countries: A position statement for the International Society for Infectious Diseases	Laboratory surveillance system not highlighted
16.	Implementation of the world health organizationâ€™s global antimicrobial resistance Surveillance System (GLASS) for the surveillance of sputum specimens collected from patients at Siriraj Hospital	Full-text article not accessible
17.	Laboratory diagnosis of novel corona virus (2019-nCoV)- present and the future	Laboratory surveillance system not highlighted
18.	Mortality from external causes in Africa and Asia: evidence from INDEPTH Health and Demographic Surveillance System Sites	Laboratory surveillance system not highlighted
19.	Mortality Surveillance Methods to Identify and Characterize Deaths in Child Health and Mortality Prevention Surveillance Network Sites	Laboratory surveillance system not highlighted
20.	Multicenter, hospital-based surveillance of rotavirus disease and strains among indian children aged <5 years	Year of publication not as per inclusion criteria
21.	Multisite virological influenza surveillance in India: 2004-2008	Year of publication not as per inclusion criteria
22.	Pathology and Telepathology Methods in the Child Health and Mortality Prevention Surveillance Network	Laboratory surveillance system not highlighted

23.	Poliovirus Laboratory Based Surveillance: An Overview	Full-text article not accessible
24.	Pregnancy-related mortality in Africa and Asia: evidence from INDEPTH Health and Demographic Surveillance System sites	Laboratory surveillance system not highlighted
25.	Public Health Laboratory Surveillance and Diagnosis of Japanese Encephalitis: Time to Revisit	Laboratory surveillance system not highlighted
26.	Specialist laboratory networks as preparedness and response tool - the Emerging Viral Diseases-Expert Laboratory Network and the Chikungunya outbreak, Thailand, 2019	The laboratory network not part of WHO SEARO region.
27.	Standardization of Minimally Invasive Tissue Sampling Specimen Collection and Pathology Training for the Child Health and Mortality Prevention Surveillance Network	Laboratory surveillance system not highlighted

Table S2: List of Surveillance System for which websites/ grey literature was not accessible.

S.No.	Name of the Surveillance system identified within the articles
1	National Rotavirus Surveillance Network(NRSN)- India
2	Health and Demographic Surveillance sites(HDSS)
3	Invasive Bacterial Infection Surveillance’(IBIS)
4	Alliance for Surveillance of Invasive Pneumococci’ (ASIP),
5	Pan Asia Epidemiologic Surveillance Network
6	Hospital Based Sentinel Surveillance for Bacterial Meningitis (HBSSBM)
7	Hospital-based sentinel surveillance for Streptococcus pneumoniae and other invasive bacterial diseases in India (HBSSPIBD)-India
8	WHO Invasive Bacteria -Vaccine Preventable Disease Surveillance Network
9	Hybrid AMR Surveillance system
10	AIV surveillance system
11	Severe Pneumonia (SevPn) surveillance system
12	National Meliodosis Surveillance Program

13	Bangladesh, Bhutan, India, Nepal, Sri Lanka Malaria Drug Resistance Network (BBINS)

Table S3: Characteristics of studies included

S.No	Author, year	Design of study	Aim and objective of study	Study Location	Characteristics of Surveillance						Diseases Captured	Gaps and Challenges identified in the system	Genomic Surveillance component
					Name of surveillance system	Type of surveillance<br>1.Active<br>2.Passive<br>3.Sentinel<br>4.Special Surveys	Level of surveillance<br>1.District<br>2.State<br>3.National	Mode of surveillance<br>1.Paper based<br>2.Electronic	Type of Surveillance system<br>1.Vertical<br>2.Integrated	Laboratory component			
1.	Gandra et al, 2020	Review	To discuss health systems, laboratory	South and southeast Asian	AMR Surveillance	Passive surveillance of laboratory data.	NR	Paper-based	NR	Thailand comprises a comprehensive laboratory	Antimicrobial Resistance	Most nations, have inadequate infrastructure and	NR

			capacity, bacterial disease burden, AMR surveillance progress, status, challenges, opportunities and progress in efforts to tackle AMR in eight South and Southeast Asian	countries.						network and capacity.<br>All the countries have national reference laboratory selected for AMR surveillance.<br>Private sector Laboratories are more compared to public sector.		laboratory capacity in the public sector<br>There are no accredited laboratories meeting international standards in Nepal.<br>It is difficult to gather laboratory data electronically, and IT usage is modest.	

			countries .							Only Thailand has comprehensive AMR surveillance system and plans of further expanding it.			
2.	May et al, 2011	Review	To demonstrate syndromic surveillance systems are being used in low-	Southeast Asian (India, Thailand) Western pacific	Syndromic Surveillance	Syndrome based surveillance	India: state	India: Both Thailand: Both	Integrated disease surveillance system in India	Laboratory used for diagnostics	India: acute flaccid paralysis, acute dysentery, cholera-like disease, diphtheri	Personnel related challenges in the form of lack of knowledge, training, resulting in delayed reporting.	NR

			resource settings	Eastern Mediterranean<br><br>Regions of Americas<br><br>African countries							a, encephalitis, fever with bleeding tendency<br><br>Thailand: focused on constitutional, respiratory, and gastrointestinal syndrome		
3.	Mercado et al, 2017	Cross-sectional	study assessed the current sources	22 target countries in the	National malaria control program	NR	NR	15 countries mentioned they are in	NR	Data collected from	Malaria	Engaging private sector is a challenge	NR

			of malaria surveillance data collected by NMCPs and the role of the private sector in malaria treatment in the Asia Pacific region.	Asia Pacific: Afghanistan, **Bangladesh, Bhutan**, **Democratic People's Republic of Korea** (DPR Korea), **India, Indonesia, Malaysia, Myanmar**, **Nepal**, Pakistan, **Sri Lanka, Thailand,**	(NMCP) surveillance system.			the process of rolling out electronic data reporting system.<br><br>7 countries mentioned they have established electronic reporting system as a primary medium for reporting.<br><br>5 countries did not specify		laboratories		in many countries.	

				**Timor-Leste**,				their reporting method.					
4.	Phalkey et al, 2013	Multicentric retrospective cross-sectional evaluation study.	study aimed to assess the structure and performance of the Integrated Disease Surveillance System. Also assessed challenges in the system.	India	IDSP	Involves both active and passive surveillance.	All the three levels.	Both	Integrated	Through IDSP, a referral network of ten labs was formed in the state, however it is not operating at its best.	Multiple diseases	Challenging support activities including lab, communication tools, transportation, training, and other tools.<br><br>Sometimes reporting quality is	NR

												compromised.<br><br>Issues with logistics resulting from the storing and transport of huge amounts of printed formats.	
5	Lim at al, 2020	Not identified	study aimed to develop and test an application that can support a	People's Democratic Republic, Myanmar, Nepal, Thailand, the and	Antimicrobial resistance surveillance system (AMASS)	NR	NR	Offline Electronic data collection.	NR	Medical facilities with a microbiology lab.<br><br>With the least amount of	AMR	NR	NR

			local hospital to analyse routinely collected electronic data independently and generate AMR surveillance reports rapidly	Vietnam						resources and skills needed, the AMASS can assist hospitals with microbiology laboratories in evaluating frequently collected data and producing reports. This may give individual hospitals the power to improve			

										local knowledge of AMR, take appropriate action, and make the most of available local data.			
6	Setiawaty et al, 2014	Primary study: Outbreak investigation report	Study was conducted to discuss the outbreak investigation experiences and	Indonesia	Event based surveillance<br><br>District surveillance officer (DSO) surveillance	Active, passive and sentinel.	Involves all the three levels.	NR	NR	The national influenza centre and laboratory network for influenza were built to hasten the	Avian influenza ::H5N1	Underuse of the tools and reagents for diagnosis.<br><br>Management of inventories and	Genetic characteristic of virus were taken into consideration during outbreak investigation.

		Cannot be categor ized	ways to improve the process in the future.		system for active case detection .<br><br>Rumour surveillan ce<br><br>Sentinel influenza like illness (ILI) surveillan ce<br><br>Severe Acute Respirato ry Illness (SARI)					influenza A (H5N1) response.<br><br>Animal disease detection facility for the region.<br><br>These laboratory networks may one day be used to diagnose not only influenza but also other new infectious diseases.		upkeep of laboratory equipment are problems due to a lack of qualified laboratory personnel.<br><br>Other difficulties experience d by the network included inadequate supplies of equipment and reagents in other	

					surveillance					In Indonesia, 44 labs were designated as the H5N1 influenza A diagnostic laboratories. They were spread across 21 of Indonesia's 33 provinces. Eight regional labs in eight provinces,		laboratories.	

										34 subregional labs in thirteen provinces, and two referral laboratories in Jakarta.			
7	Jayaraman et al, 2018	Surveillance study<br>Cross-sectional	To describe the framework of the Hospital Based Sentinel Surveillance for Bacterial Meningiti	India	Hospital based sentinel surveillance for bacterial meningitis	Sentinel	NR	Electronic data transfer using Epi-Info on a monthly basis.<br>Hard copies of the CRFs were	NR	Christian Medical College, Vellore serves as the Reference Laboratory for the network and administered external quality assurance	Bacterial meningitis	NR	NR

			s (HBSSBM) network in India and findings from the surveillance activity carried out during 2012–2013.					received at the coordinating center for second data entry, data validation, and analysis		(EQA) programme for the participating sentinel sites<br>External quality evaluation done by reference laboratory.			
8	Bose et al, 2013	Report<br>Case Based Surveill	The paper describes the epidemiology of measles	Pune, India	Measles Surveillance System	This is the first report from an active case based laboratory supported	State and district. No National system for case based laboratory supported	Case reporting use Case investigation form (CIF)	NR	Laboratory supported surveillance system	Measles	NR	NR

		ance study<br>Not clearly identifi ed	in three MAVP blocks of Pune for the period Novembe r 2009 through Decembe r 2011.			measles surveillanc e system in India.	measles surveillanc e system.						
9	Sathku mara et al, 2018	Report<br>Not clearly defined	To develop a phylogeo graphic appreciat ion of disease emergen ce, understa nd its	Sri Lanka	National surveillan ce program me	NR	National	NR	NR	Reference laboratory supported surveillanc e	Clinical melioidos is	NR	NR

			progression to different clinical outcomes, and measure the extent of disease class/genotype correlation										
10	Capeding et al, 2013	prospective, multicenter, active fever surveillance,	To determine the incidence and proportion of acute	Indonesia, Malaysia, Philippines, Thailand and	NR	Active surveillance conducted for this study	District	Electronic	NR	Case confirmation and testing	Dengue, chikungunya, hepatitis A and influenza A viruses, leptospir	NR	NR

		cohort study	febrile episodes that were caused by dengue, as well as by chikungunya, hepatitis A and influenza A viruses, leptospirosis, rickettsia, and S.Typhi	Vietnam							osis, rickettsia, and S.Typhi		
11	Kaur et al, 2019	Review	Discusses the develop	India	Hybrid AMR Surveilla	NR	National	Data on the results of the	NR	Collect data from various	AMR	NR	The platform is also

			ment of i-AMRSS by the Indian Council of Medical Research (ICMR) for the management and analysis of AMR data from different laboratories.		nce system: Antimicrobial resistance surveillance system (i-AMRSS)			Antimicrobial Susceptibility Testing (AMST) are collected electronically through a web portal. In order to have standardised data, the system provides built-in reporting criteria or distinct stakeholders.		laboratory network			designed to facilitate genomic studies by identifying priority resistant pathogens

12	Bhunti et al, 2019	Surveillance study Report<br><br>Potential cross-sectional	This report describes the methodology of the SevPn surveillance system and provides preliminary results on each of its objectives from December 2010 through	Thailand	Severe Pneumonia (SevPn) surveillance system	Passive and sentinel	Surveillance and Rapid Response Teams' (SRRT) at national, district, provincial and community level.	Electronic i.e. web based system	NR	Comprises of a laboratory network	Severe Pneumonia	Inadequate laboratory detection abilities.<br><br>86% of prospective cases discovered during surveillance audits not submitted to the SevPn system.	NR

			December 2015										
13	Murhekar et al, 2019	Secondary study	Analysed the laboratory surveillance data from 51 functional VRDLs collected from January 2016 to July 2018 to describe the epidemiology of	India	NR	sentinel	All levels	Web-based data entry system	NR	consists of the Network for Virus Research and Diagnostic Laboratory (VRDLN). Regional biosafety level 3 laboratories and state and medical college biosafety level 2	Chikungunya	NR	NR

			CHIKV in India.							laboratories are among the labs in the network. All standard and specialised testing are available in local laboratories.			
14	Murhekar et al, 2019	Report<br><br>Potential Cross-sectional	analyzed the laboratory surveillance data from 2014 to	India	NR	Sentinel	All levels	Both	NR	Comprises of Virus Research and Diagnostic Laboratory Network (VRDLN).	Dengue	NR	NR

			2017 to describe the epidemiology of dengue fever in India							The network has 52 laboratories covering 24 indian states as of 2017.			
15	Setiawaty et al, 2012	Review	A review on establishing a reliable laboratory network for influenza A diagnosis during	Indonesia	NR	Sentinel	Province, district and national	NR	NR	Eight regional labs in eight provinces, 34 subregional labs in thirteen provinces, and two referral laboratories in Jakarta (NIHRD MoH/NIC and Eijkman	Influenza	Underutilisation of stocks and regents.<br><br>Lack of skilled personnel.<br><br>Insufficient equipment and reagents stocks	NR

			the avian flu (H5N1) outbreaks							Institute) make up the organisation of the laboratories.<br><br>To ensure quality, the laboratories conduct regular quality inspections.<br><br>Regular training in biosafety and biosecurity is also conducted.			

16	Manurang et al, 2021	Qualitative study design	This study used well-established guidelines to evaluate the operation of EWARS in Indonesia's West Papua province.	Indonesia	Early Warning and Response System (EWARS)	NR	NR	The source data is reported by surveillance officers using an automated data collection method called SMS reporting. Health professionals start gathering data at the sub-district level and provide it to the	NR	Comprises of national reference laboratory	NR	Mobile and internet network connectivity limited human and funding resource Data reporting is delayed sometimes. Need for laboratory strengthening.	NR

								surveillance officer via SMS, phone, or hand delivery. The officials then compile all the data from the district and send 1 SMS to the application programming interface (API) of the national Ministry of Health in					

								the main EWARS computer system. The central EWARS database receives the data via the API, where they are automatically aggregated, examined, and reported on the main EWARS website.					

17	Cavallaro et al, 2016	Surveillance study<br><br>Cannot be defined clearly	The purpose of this paper is to evaluate the feasibility of expanding polio–measles surveillance and laboratory networks to detect bacterial meningitis and JE, using surveillan	India, Bangladesh and China	NR	India- Sentinel<br><br>Bangladesh- Survey	National, district	In India and Bangladesh, polio-measles surveillance employees employed the software programme Epi InfoTM (CDC, Atlanta, USA).<br><br>Results reporting is more reliable for reporting	NR	Due to differences in laboratory staff and viral and bacterial testing procedures, polio-measles laboratory networks were helpful for developing JE diagnostic capacity but not for confirming	Bacterial meningitis and JE	For laboratory data, data management and reporting are poorer.<br><br>No countries fared well when CSF samples were sent to secondary and tertiary laboratories due to inadequacies in tools, training,	NR

NR

			ce for acute meningitis-encephalitis syndrome in Bangladesh and China and acute encephalitis syndrome in India					clinical cases but less reliable for reporting laboratory data.<br>Laboratory data is manually entered in Bangladesh, whereas it is entered into spreadsheets in India.		bacterial meningitis.		and standards for quality assurance and control.	

18	Sirivongrangson et al, 2018	Surveillance study<br><br>Potential Cross-sectional	This manuscript describes the first year of EGASP surveillance in Thailand during November 2015 – October 2016 and are important for early detection of NG resistant strains	Thailand	Enhanced Gonococcal Antimicrobial Surveillance Programme (EGASP)	Sentinel	NR	both	NR	Comprises of 2 reference laboratories for syndromic data and case confirmation	Antimicrobial-resistant Neisseria gonorrhoeae	NR	NR

			and informing treatmen t options										
19	Rajkum ar et al, 2020	cross-section al multi-site hospital - based surveil-lance study	To describe the framewo rk and protocol of the ongoing multi-site HBSSPIB D study	India	hospital-based sentinel surveillan ce for S. pneumon iae and other invasive bacterial diseases (HBSSPIB D)	sentinel	all	The study used paper-based Case Report Forms (CRFs) to gather its data. The lab personnel received training on CRF forms and reporting in the	NR	. Induction training on laboratory practises for sample collection, transporta tion, processing in the laboratory, testing and document ation, as well as laboratory-based case recruitmen	S. pneumon iae	NR	NR

								ICMR-NIE online data entry and management system.		t, was provided to laboratory investigators and project laboratory employees at the National Reference Laboratory (NRL).			
20	Herini et al, 2017	Cross-sectional study	A prospective surveillance study of infants aged <1 year with	Indonesia	Congenital rubella surveillance system	NR	NR	NR	NR	For case conformation	Congenital Rubella Syndrome	NR	NR

			suspected CRS.										
21	Sirijatuphat et al, 2018	Cross-sectional may be?	Reported the results of implementing GLASS for surveillance of blood culture specimens in patients who had their blood culture samples collected	Thailand	Global antimicrobial resistance surveillance system (GLASS)	NR	NR	Web based application was used for collecting patient data for this study	NR	GLASS was found Superior to laboratory based surveillance system	AMR	GLASS was found to be more time consuming and required more resources.	NR

			for clinical purposes										
22	Moreno et al,2019	prospective, active surveillance,hospital-based epidemiological study.<br><br>Not clearly identified	This study aims to determine the frequency of influenza related hospitalisations in different countries, by circulating strains and age groups, to study	St. Pet Moscow Kazakhstan Czech Rep. Canada Romania Turkey Valencia Tunisia Suzhou /Shanghai India Mexico	Global Influenza Hospital Surveillance Network (GIHSN)	active	NR	NR	NR	Case conformation	Influenza	NR	NR

			risk factors for influenza - associate d hospitalis ations and estimate Influenza Vaccine Effective ness (IVE) by site, age group and strain	SouthAf rica									

23	Kant et al, 2010	Review	Use of information and communication technology in disease surveillance	India	IDSP	NR	All levels	Both, through forms and Web portal	Integrated	Laboratory based surveillance in which the laboratory is supposed to report confirmation of cases using L forms	Infectious diseases	NR	NR
24	Naser et al, 2015	Cluster and case based Surveillance study	This paper explores the utility of cluster- and case-based	Bangladesh	NR	Active and passive	District	NR	NR	Case detection and confirmation	Nipah	NR	NR

		Crossectional	surveillance established in government hospitals in Bangladesh to detect Nipah virus, a stage III zoonotic pathogen.										
25	Saha et al, 2017	Cross sectional surveill	Aimed to evaluate the logistics, cost and	Bangladesh	Invasive Bacterial-Vaccine Preventable	Active and sentinel	NR	NR	Integrated	Case conformation and	Invasive Bacterial-Vaccine Preventable	NR	NR

		ance study??	sustainability of leveraging the ongoing WHO-coordinated IB-VPD platform for enteric feversurveillanceby broadeningtheinclusioncriteriaofthe originalsurveillance		Diseases (IB-VPD) Surveillance System.					surveillance	Diseases-Enteric fever		

26	Manoharan et al, 2016	prospective hospital-based and retrospective laboratory-based surveillance study<br><br>Not clearly defined	To provide nationally representative data for the pattern of disease due to S pneumoniae, current trends in the serotype of invasive pneumococci, and invasive	India	NR	Sentinel	NR	NR	NR	Case confirmation and surveillance	Invasive pneumococcal disease	NR	NR

			pneumococci antimicrobial resistance patterns, in India										
27	Ashley et al, 2018	Review	To summarize the supranational surveillance networks for drug-resistant infections operating in LMICs	LMICs	Global Antimicrobial Resistance Surveillance System (GLASS)	Active, passive and Sentinel	NR	NR	NR	Comprises of laboratory network for surveillance	AMR	Small coverage<br>Insufficiently representative data<br>Implementing diagnostic pauses in clinical practise	NR

			since 2000 and dis-cuss their impacts and challenges, and any implications for the implementation of GLASS.										might be challenging. Inactivity on the part of partners Delays in reporting Understaffing and underfunding are not prioritised.	
28	Saha et al, 2016	Population Based surveillance study	Aims to determine the aetiology of neonatal infections in 5 populatio	Bangladesh, India and Pakistan	Aetiology of Neonatal infection in South	NR	NR	Electronic: ANISA data capture forms (DCFs)-laboratory	NR	Using an electronic laboratory data capture system for diagnostics	Neonatal infections	Specimen collection and transport to designated study	NR

		Not clearly identified	n-based sites in Bangladesh, India and Pakistan.<br><br>In this article, we describe the laboratory methods of ANISA, including the rationale for molecular platform selection and the associated challenges of		East Asia (ANISA)			data system		To record the pertinent information about each step of the specimen processing procedure, ANISA data capture forms (DCFs) were developed.<br><br>These DCFs can take the role of conventional laboratory notebooks; built-in checks in the data entry system reduce the		laboratories and timely processing in rural settings<br><br>Minimal or non-existent laboratory facilities at the field sites;	

			implementing this testing in difficult field settings. We also discuss measures taken to improve the detection of a wide range of bacteria and viruses through molecular testing of blood and respiratory specimens along with the complementary tradition							likelihood of transcribing errors.<br>The lab staff conducting the tests enters all laboratory data on DCFs into the ANISA databases at the individual sites in real time.<br>Weekly data transfers from all stations in Dhaka, Bangladesh, to the			

			al culture methods employe d							main data server.			
29	Tissera et al, 2016	Cross section al surveill ance study	we describe the study sites, research design and the findings of the first two years of laborator y-enhance d sentinel surveillan	Sri Lanka	laborator y-enhance d sentinel surveillan ce system	Active, passive and sentinel	National and district	Case reporting forms used to collect data	NR	The project chose the Genetech Research Institute (GRI) Colombo as a supporting reference laboratory, and during the project's first year, GRI	Dengue	NR	NR

			ce system							Colombo served as an interim testing laboratory.			
30	Bala et al, 2013	Retrospective study-Secondary study	This study was conducted to analyse the AMR profile of N gonorrhoeae in selected SEAR countries, based on retrospec	6 SEAR countries (namely Bhutan, Indonesia, India, Myanmar, Sri Lanka and Thailand)	NR	NR	NR	NR	NR	In 1997, WHO GASP was founded in the South-East Asia Region (SEAR). The Safdarjung Hospital's Regional STD Teaching, Training and Research	AMR	NR	NR

			tive AMR data communicated by the focal point laboratories participating in the WHO GASP during the period 2009 to 2012.							Center in New Delhi, India, was acknowledged as the regional reference laboratories (RRL)			
31	Saluja et al, 2016	Observational study	to carry out a hospital-based surveillan	India	Hospital based surveillance	NR	NR	Case reporting form from	NR	Case detection	Gastroenteritis	NR	All stool samples that had rotavirus VP6

		Prospective Hospital Based Surveillance<br><br>Cross-sectional	ce of rotavirus gastroenteritis in children≤ 59 months of age and develop estimates of disease burden in the population under surveillance					hospital log books					antigen results that were positive underwent strain characterisation by ELISA. At the Central Laboratory, reverse-transcription polymerase chain reaction was used for genotyping (RT-PCR)

32	Shakya et al, 2012	Not clearly identified	To analyse the biochemical characteristics, antimicrobial susceptibility trend, genetic relatedness and the ctxB type of V. cholerae strains isolated during 2007–2010 at	Nepal	national antimicrobial resistance surveillance network	NR	All the levels	NR	NR	The 10 network laboratories that conduct the ongoing national antimicrobial resistance (AMR) surveillance include the National Public Health Laboratory of Nepal (NPHL)	AMR	NR	Genotypically identified Vibrio cholerae were found in ten different hospital laboratories in Nepal between 2007 and 2010 from patients who had diarrhoea.

			different parts of Nepal										
33	Kumar et al, 2016	Report<br><br>Potential cross-sectional	This preliminary report describes rotavirus burden in children admitted with acute gastroenteritis between September 2012 and<br><br>December 2014 by age, region, seasons and also	India	Indian National Rotavirus Surveillance Network	NR	National and district	At each of the regional and referral laboratories, data were input into the CRF using the online data collecting portal that is hosted on the NIE web server.	NR	Comprises of referral laboratories	Acute gastroenteritis	NR	Rotavirus positive samples were used to perform reverse transcription polymerase chain reaction (RT-PCR) rotavirus genotyping for VP7 (G-typing) and VP4 (Ptyping).

			the diarrheal disease severity pattern										
34	Turner et al, 2013	Hospital case based surveillance study<br><br>Potential cross-sectional	The aim of this laboratory-enhanced, hospital-based surveillance was to characterise the epidemiology of respiratory virus infection	Thailand-Myanmar border	NR	NR	NR	Case record forms	NR	Case detection and surveillance	Respiratory virus infections	Lack of proper data representativeness.	NR

			s among refugees living on the Thailand-Myanmar border.										
35	Murhekar et al, 2018	Hospital based sentinnel Surveillance report<br>Report	This is the first report of long-term CRS surveillance data in India	India	laboratory-supported surveillance for CRS(Congenital Rubella Syndrome)	Sentinel	NR	NR	NR	Lab Diagnosis	Congenital Rubella Syndrome	NR	Oropharyngeal swabs should be taken from all infants who are 6 months old at the time of enrollment and transferred to the National Institute of

													Virology in Pune for RT-PCR testing and genotyping in accordance with WHO standards.
36	Corea et al, 2016	Laboratory Based Case finding program<br><br>Not clearly defined	To describe the results of that laboratorybased case-finding program: the geographic	Srilanka	National Meliodosis Surveillance Program	NR	national	electronic	NR	Coordinated by the Western Australian Public Health Laboratory, lab diagnosis<br><br>Clinical laboratory molecular technology was first	Invasive Melioidosis	NR	MLST Genotyping done and Coordinated by Western Australian Public Health Laboratory

			distributi on of invasive melioidos is in Sri Lanka, the principal epidemio logic risk groups,							brought to Sri Lanka with a deployable molecular laboratory as part of the World Health Organizati on's laboratory capability-building programm e, and since then, a nationwide melioidosis monitoring programm			

										e has been developed.			
37	Aparna Shah, 2017	Review	To suggest actions for policy makers to adopt as part of their national action plans to establish robust nationwide laboratory based surveillance	Sourh East Asia Countries	NR	NR	AMR surveillance is limited to national or regional laboratories, leaving the district laboratories out of any surveilla	NR	NR	In 2015, a WHO examination of the regional situation revealed that, of the region's 11 member nations, six have national policies or strategies on AMR, seven have national coordinating mechanisms, and nine have regulatory agencies	AMR	Poor infrastructure for lab based surveillance.	NR

			mechanis ms for AMR.				nce net work."			to monitor AMR. 4 The state of laboratory-based surveillanc e is impacted by each of these variables. Six of the national reference laboratorie s for antibiotic sensitivity testing take part in external quality assessmen t programm es. and seven said that their entire network of			

										tertiary hospitals is supported by a strong laboratory infrastructure. Six nations take part in external quality assessment programmes, while nine nations have national reference laboratories for determining antibiotic sensitivity. There is no information available about how many laboratorie			

										s adhere to international standards.<br><br>There is a dearth of trustworthy and comparable data since standards from organisations like the Clinical and Laboratory Standards Institute and the European Committee on Antimicrobial Susceptibility Testing are not frequently			

										employed. Standards for surveillance are not universally accepted in all nations. National data are frequently restricted to proportions of resistant bacteria, even though reporting the percentage of resistant bacteria causing specific diseases or affecting identified			

										groups is preferred. Despite the software's free availability and ease of use, WHONET is not frequently used to track patterns of resistance. According to data acquired by the WHO regional office, the infrastructure and general state of the nation's health laboratorie			

										s have an impact on how well surveillance is conducted.			
38	Garg et al, 2018	Review	The aim of this paper was to assess	India, Nepal, Bangladesh, Myanm	NR	NR	NR	NR	NR	By leveraging the capabilities of a few	Polio, AFP	Inconsistent and incomplete data from all	Two facilities in India (Hyderabad and

			the supplementary role of ES in the ongoing AFP surveillance and highlight the current challenges in expansion of ES in the WHO South-East	ar, Thailand						chosen polio laboratories from the Global Polio Laboratory Network's 16 existing facilities in the area, laboratory support for ES was established.		administrative levels,	Patna) and one in Nepal also do sewage concentration solely, and the laboratories support viral isolation, intratypic discrimination, and genomic sequencing of poliovirus isolates from sewage sludge.

39	Njelesani et al, 2014	Qualitative and Quantitative assessment study<br><br>Mixed method study	To use available evidence to describe the optimal capacities needed at each of laboratory if they were to achieve the goal.<br><br>To develop a set of assessment and monitoring collection tools that would enable us to assess what	Sri Lanka	NR	NR	NR	NR	NR	The laboratory has a respectable workforce, access to research funding, and regional and national partnerships.	Lymphatic Filariasis	Financial issues for NTD research.<br><br>Absence of safety mechanisms and paperwork for quality assurance<br><br>.	NR

			capacity gaps needed addressing if laboratories were to achieve optimal capacity and<br><br>To develop a capacity strengthening action plan to address the gaps and indicators that would enable us										

			to monitor progress as capacity gaps were addressed										
40	Vijay et al, 2021	Cross-sectional study	to assess the preparedness of veterinary laboratories in India to participate in an integrated	India	NA	NA	NA	NA	NA	established animal laboratories for AMR surveillance, although these laboratories are not currently integrated with the human.	AMR	NR	NR

			antimicrobial resistance surveillance network							The veterinary and environmental sectors have limited capability for antibiotic susceptibility testing, human sources			

Table S4: List of Surveillance system found in the studies

S.No.	Name of Surveillance system	Country	Type of surveillance<br><br>1.Active<br><br>2.Passive<br><br>3.Sentinel<br><br>4.Special Surveys	Level of surveillance<br><br>1.District<br><br>2.State<br><br>3.national	Mode of surveillance<br><br>1.Paper based<br><br>2.Electronic	Laboratory component	Diseases Captured
1	Global Antimicrobial Surveillance System (GLASS)	All southeast Asian countries are participants along with other countries from different region.	sentinel	Local to country-level data collection	According to the national AMR surveillance system protocol, participating surveillance sites regularly submit data to the NCC using data management software or paper forms. NFPs are required to submit aggregated AMR data for the prior year as	NRLs provide information on isolates from over 700 sentinel laboratories spread across the member nations.<br><br>When isolates have atypical kinds of AMR, the network laboratories issue an alarm (referred to as "event-driven surveillance" here).	AMR

					well as details about the status of their surveillance system to the GLASS-IT platform.<br><br>Once a year, GLASS requests data from countries.	The quality of test reagents and test performance are ensured by a number of standard assessment processes in each of the participating sentinel laboratories. Each NRL acts as an external quality control programme to guarantee the performance standards of sentinel laboratories around the country.	
2	Child Health and Mortality Surveillance Network(CHAMPS)	Bangladesh, Ethiopia, Kenya, Mali, Mozambique, Sierra Leone, and South Africa	Local to national	NR	NR	Pathology and High Consequence Pathogens Division The major pathology lab for CHAMPS is DHCPP's Infectious Disease Pathology Branch, which examines specimens by histology for signs of infectious or other disorders and conducts tissue-based diagnostic tests.	causes of stillbirth and death in children under five
3	National Rotavirus Surveillance Network(NRSN)	India	Website not found				
4	Health and Demographic	Grey Literature					

	Surveillance sites(HDSS)	not found					
5	National Antibiotic Surveillance Network	Nepal	NR	Local to national level	NR	The National Public Health Laboratory (NPHL) has evolved with the current network of 20 laboratories encompassing all five regions of the nation, and it has added eight pathogens of relevance.	AMR
6	Global Influenza Hospital Surveillance Network (GIHSN)	India Nepal and other 16 countries	Active and special survey*	Local to national	NR	Samples sent to reference laboratories	Influenza
7	Invasive Bacterial Infection Surveillance'(IBIS)	No grey literature found					
8	Alliance for Surveillance of Invasive Pneumococci' (ASIP),	No grey literature found					
9	Asian Network for Surveillance for Resistant	14 Asian Countries- India, Indonesia, Malaysia, Korea,	Multiple study surveillance study	Local to country	NR	NR	AMR

	Pathogens'	Thailand, and Srilanka (searo)					
10	Pan Asia Epidemiologic Surveillance Network	No Grey literature found					
11	Antimicrobial resistance Surveillance System (AMASS)	NR	NR	NR	Electronic	Microbiology data from hospitals are transferred to the system	AMR
12	Hospital Based Sentinel Surveillance for Bacterial Meningitis (HBSSBM)	No Grey literature found					
13	Enhanced Gonococcal Antimicrobial Surveillance Programme (EGASP)	WHO- GLASS member states	Sentinel	Country-level	A standardized abstraction form will be used to record information about the fields (as developed by the EGASP country).<br>The EGASP samples and these results will subsequently be sent	EGASP reference laboratories assist EGASP sentinel clinics by performing culture and antibiotic susceptibility tests in accordance with the EGASP methodology and laboratory SOPs.	Gonococcal Antimicrobial Resistance

					to the reference laboratory (s).<br><br>The EGASP coordinator must receive all patient and laboratory data no later than two months following the end of the month in which the corresponding isolates were obtained.<br><br>Utilizing information technology solutions that have already been adopted and put into place at the national level, data can be digitalized.<br><br>(Paper as well as electronic)		
14	Hospital-based sentinel surveillance for Streptococcus pneumoniae and other invasive bacterial diseases in India (HBSSPIBD)	India	No grey literature found				

15	WHO's Gonococcal Antimicrobial Surveillance Programme (GASP)	India, Indonesia, Malaysia, Sri Lanka, Thailand and other countries	NR	Regional to country	NR	Regional coordinating centres oversee the GASP global network of laboratories. VMMC and Safdarjang Hospital are the WHO GASP South-East Asia Regional Reference Laboratory locations in New Delhi, India.	Gonococcal antimicrobial resistance
16	WHO Invasive Bacteria -Vaccine Preventable Disease Surveillance Network	No grey literature found					
17	WHO Global Influenza Surveillance and Response System (WHO GISRS	123 WHO Member States:	Active global system	Regional to country level	Report weekly to flunet	Comprises of 4 Essential Regulatory laboratories (ERL) and 13 WHO H5 Reference Laboratory	Influenza
18	Hybrid AMR Surveillance system	No grey Literature found					
19	AIV surveillance system	No Grey Literature found					
20	Severe Pneumonia (SevPn) surveillance system	No grey literature found					
21	National Meliodosis	Srilanka	No Grey Literature				

	Surveillance Program		found				
22	Indian network for surveillance of AMR.	India	NR	Regional to national level	real time online AMR data entry system	The network consists of 16 regional centres (RC). As part of a nationwide network known as Regional Centers (RCs), sixteen regional labs from tertiary care hospitals are supplying data on a set number of isolates for each pathogenic group.	Gram negative non-fermenters, enteric fever pathogens, diarrhea-causing bacteria, staphylococci and enterococci, as well as yeasts (Candida and Cryptococcus spp.) and mycelial fungus (not included in the list of WHO priority

							pathogens) (Aspergillus spp. and Zygomycetes spp.).
23	Bangladesh, Bhutan, India, Nepal, Sri Lanka Malaria Drug Resistance Network (BBINS)	Bangladesh, Bhutan, India, Nepal, Sri Lanka	No Grey Literature found				

Table S5: Modifications during COVID-19

S.No	Author, year	Design of study	Aim and objective of study	Study Location	Characteristics of Surveillance system						Diseases Captured	Gaps and Challenges identified in the system	Modification in the existing system
					**Name of surveillance system**	**Characteristics of surveillance system: 1. Type of surveillance 1.Active 2.Passive 3.Sentinel 4.Special Surveys**	**Level of surveillance 1.District 2.State 3.national**	**Mode of surveillance 1.Paper based 2.Electronic**	**Type of Surveillance system 1.Vertical 2.Integrated**	**Laboratory component**			
1.	Potdar et al, 2020	descriptive epidemiology of COVID-19 cases by time,		India	COVID-19 Surveillance	Sentinel surveillance for severe acute respiratory infection (SARI)	All the three	Electronic	NR	Indian Council of Medical Research (ICMR) has been leading India's laboratory surveillance	COVID-19	missing data was high	The testing centres complemented the IDSP and the State Public

		place and person								testing for COVID-19. In the initial phase, testing for SARS-CoV-2 was conducted through 78 selected national reference laboratories. The ICMR led the expansion of testing capacity by using its existing laboratory network, developing standard protocols and launching an online portal for reporting			Health Departments in an effective way to augment the testing capacities for COVID-19

										The infrastructure for testing included ICMR institutes and partners through the Virus Research and Diagnostics Laboratories (VRDL) Network			
2.	Kumar et al, 2020	Review	his review summarizes various molecular diagnostics methods, technical guidelines, and advanced testing	India	NR	NR	All the three levels	Both	NR	ICMR has scaled up COVID-19 testing laboratories in partnership with DST,DBT, ICAR, CSIR, DRDO, MHRD, medical	COVID-19	NR	ICMR expanded testing laboratories

			strategies adopted in India for laboratory diagnosis of COVID-19							colleges, and private laboratories. As of May 30, 2020, there has been a total of 669 COVID-19 testing labs in India, including 466government laboratories and 203 private laboratories. Of these,480 labs are using RT-PCR-based tests, 134 labs are using TruNat tests, and 55 labs are using CBNAAT-based			

										COVID-19tests (20).			
